# Supplementary material for: Polymorphisms in Pfkelch13 domains before and after the introduction of artemisinin-based combination therapy in Southwest Nigeria
Source: PLoS One. 2025 Mar 31;20(3):e0316479. doi: 10.1371/journal.pone.0316479 (PMC11957316; doi:10.1371/journal.pone.0316479)

Samples: 14741
Bases: 592
Average spacing: 25

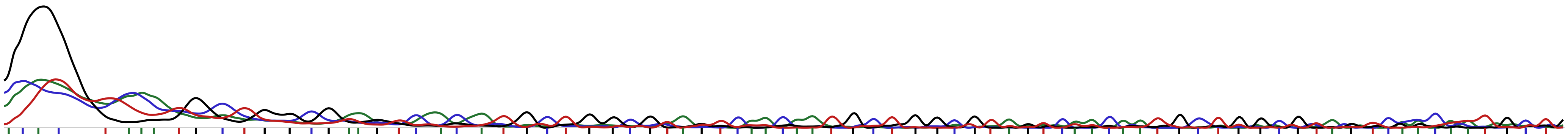

10 20 30 40 50 60 70 80

A C A C T A A A T G C T G G C G A A G T C A C A T G C T G G C G T A G T C A A C A A T G C T G G C G T A G T C A A C A A T G C T G G C G T A G T C A A C A A T G C T

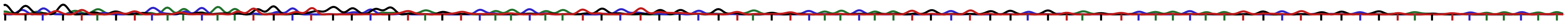

90 100 110 120 130 140 150 160

G G C G T A G T C A A C A A T G C T G G C G T A G T C A A C A A T G C T G G C G T A G T C A A C A A T G C T G G C G T A G T C A A C A A T G C T G G C G T A G T C A A C A A T G C T G G C G T A G T C A A C A A

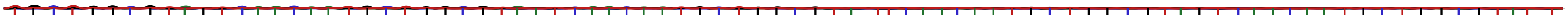

170 180 190 200 210 220 230 240 250

T G C T G G C G T A G T C A A C A A T G C T G G C G T A A T C A A C A A T G C T G G C G T A T T C A A C A A T G C T G G C G T A G T C A A C A A T G C T G G C G T A T T

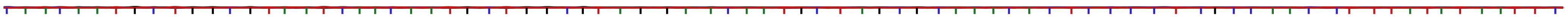

260 270 280 290 300 310 320 330

C A A C A A T G C T G G C G T A A T C A A C A A T G C T G G C G T A G G A A C A A T G C T A G C G C A A A C A C T C C C C T C G C C A A C C T T T A T A T A A T T C

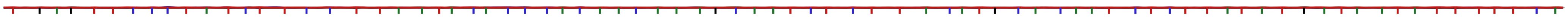

340 350 360 370 380 390 400 410

T G A G T T C C C T A T C T T C C T T A A T A C A C C C A C A A C A A A G C A A T C T C T T A C A T G C A C A A T C T C T T A T A T G A T A A T A T T T T T C A

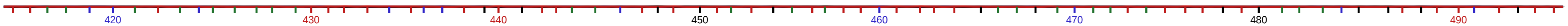

420 430 440 450 460 470 480 490

T T A A C C A T A C A A A A A A T T T T C T C C T G T G T T A A C T G T G T A T G A T A T T C T T T T G A A G A C A A T A T T T G T G A A A C G G T G T T C T G T T

Samples: 14741
Bases: 592
Average spacing: 25

500 510 520 530 540 550 560 570

G T G C T T G T T T T T G T T A A T A T G T G G T G A G G G A T A T A A A A A A A A C A T T T A A T A T G G T G A T A A A A G A C A C A C T G T G G A T T T T T C

580 590

T C C A A C C A G A G T G A T

Samples: 14741
Bases: 592
Average spacing: 25

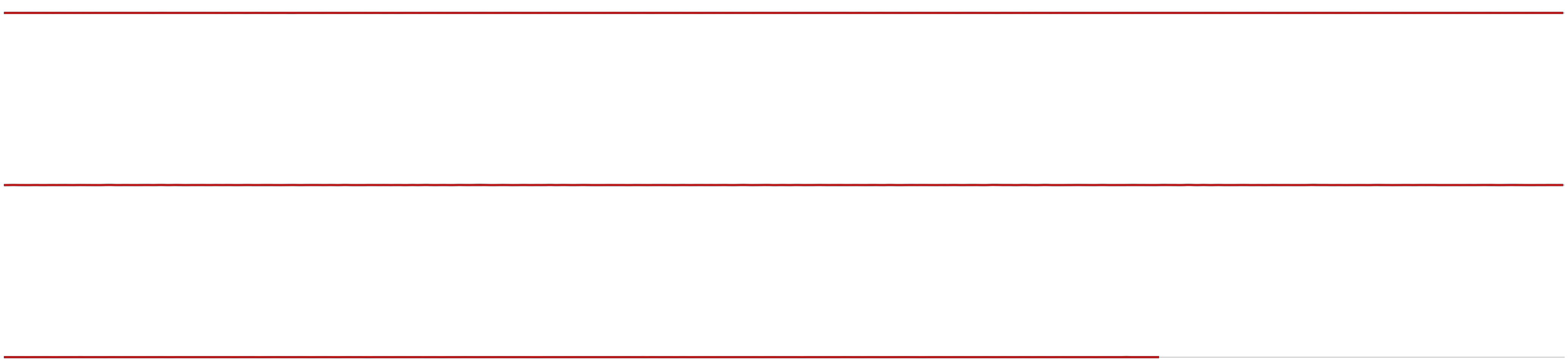

Supplement: Supporting information 2 — (ZIP) [file pone.0316479.s002.zip › 192KN2F_PREMIX_Plate_KELCH2_C07.pdf]
